# Response of High-Sensitive C-Reactive Protein to Catheter Ablation of Atrial Fibrillation and Its Relation with Rhythm Outcome

**DOI:** 10.1371/journal.pone.0044165

**Published:** 2012-08-30

**Authors:** Jelena Kornej, Claudia Reinhardt, Jedrzej Kosiuk, Arash Arya, Gerhard Hindricks, Volker Adams, Daniela Husser, Andreas Bollmann

**Affiliations:** 1 Department of Electrophysiology, Heart Center, Leipzig, Leipzig, Germany; 2 Department of Cardiology, Heart Center, Leipzig, Leipzig, Germany; University of Illinois at Chicago, United States of America

## Abstract

**Aims:**

This study investigated the possible association between hs-CRP as well as hs-CRP changes and rhythm outcome after AF catheter ablation.

**Methods:**

We studied 68 consecutive patients with AF undergoing catheter ablation. hs-CRP levels were measured using commercially available assays before and 6 months after catheter ablation. Serial 7-day Holter ECGs were used to detect AF recurrences.

**Results:**

Early AF recurrence (ERAF, within one week) was observed in 38%, while late AF recurrence (LRAF, between 3 and 6 months) occurred in 18% of the patients. None of the baseline clinical or echocardiographic variables was predictive of ERAF or LRAF. Baseline hs-CRP measured 2.07±1.1 µg/ml and was not associated with ERAF and LRAF. At 6 months, hs-CRP levels were comparable with baseline values (2.14±1.19 µg/ml, p = 0.409) and were also not related with LRAF. However, patients with LRAF showed an hs-CRP increase from 2.03±0.61 to 2.62±1.52 µg/ml (p = 0.028). Patients with an hs-CRP change in the upper tertile (>0.2 µg/ml) had LRAF in 32% as opposed to 11% (p = 0.042) in patients in the lower (<−0.3 µg/ml) or intermediate (−0.3–0.2 µg/ml) tertile.

**Conclusions:**

Changes in hs-CRP but not baseline hs-CRP are associated with rhythm outcome after AF catheter ablation. This finding points to a link between an inflammatory response and AF recurrence in this setting.

## Introduction

Local and systemic inflammation may play an important role in the initiation and perpetuation of atrial fibrillation (AF). [1]–[3] Several groups have found an association between elevated CRP levels, as an inflammatory marker, and the presence of AF. [4], [5] However, it remains still controversial whether inflammation is a consequence or one cause of AF. [6]

Multiple studies have reported that higher baseline CRP levels are associated with an increased risk of AF recurrence after electrical cardioversion [7]–[9] and that CRP decreases after cardioversion once sinus rhythm is restored. It has been shown that CRP levels are related to the left atrial size and AF duration before cardioversion, thus linking inflammation and atrial structural remodeling. [10], [11] In addition a high CRP level was associated with an abnormal left atrial substrate and a high incidence of nonpulmonary vein AF sources. [12] Moreover, one recent study has shown a relationship between local atrial inflammation and the type of AF. [13] Aside from inflammation-linked effects, CRP plays a critical role in immunity pathways, and the presence of CRP induces important phenotypic changes in the vascular endothelium, including apoptosis [14] and has direct toxic effects on endothelial cells which are mediated via reactive oxidant species. [15]

Catheter ablation has become the cornerstone of nonpharmacologic therapy of AF, with success rates generally varying from approximately 60–80%. [16] AF recurrences following ablation are frequently observed and require multiple procedures in some patients. Recently published research has suggested that AF recurrences after catheter ablation may also be related to the inflammatory response generated by the ablation procedure itself. [17], [18] Furthermore, baseline CRP was found to predict early and late AF recurrences after ablation, [19], [20] but results have not been consistent. [21], [22]

Consequently, this study investigated the association between CRP as well as CRP changes after 6 months and rhythm outcome after AF catheter ablation. It was hypothesized that the inflammatory status expressed as CRP is related with early and late AF recurrences.

## Methods

### Study Population

This study enrolled 68 consecutive patients who underwent left atrial catheter ablation for drug-refractory paroxysmal or persistent AF. In all patients, transthoracic and transesophageal echocardiography was performed prior to catheter ablation. Left atrial diameter and left ventricular ejection fraction were determined using standard measurements and a left atrial thrombus was excluded. All class I or III antiarrhythmic medications with the exception of amiodarone were discontinued at least 5 half-lives before the procedure. The study was approved by the local ethics committee (Medical Faculty, University Leipzig) and patients provided written informed consent for participation.

### Catheter Ablation

Left atrial catheter ablation was performed using a previously described approach. [23] In brief, patients were studied under deep propofol sedation with continuous invasive monitoring of arterial blood pressure and oxygen saturation. Non-fluoroscopic 3D catheter orientation, CT image integration, and tagging of the ablation sites were performed using Ensite NavX, Ensite Velocity (St. Jude Medical, St. Paul, MN, USA) or CARTO 3 (Biosense Webster, Diamond Bar, CA, USA). Trans-septal access and catheter navigation were performed with a steerable sheath (Agilis, St. Jude Medical., St. Paul, MN, USA). Patients presenting with AF at the beginning of the procedure were electrically cardioverted and ablation was performed during sinus rhythm (i.e. AF termination with ablation was not attempted). In all patients circumferential left atrial ablation lines were placed around the antrum of the ipsilateral pulmonary veins (irrigated tip catheter, pre-selected tip temperature of 48°C, and maximum power of 30–50 W). In patients with persistent AF, additional linear lesions were added at the left atrial roof, the basal posterior wall and the left atrial isthmus. Ablation of complex fractionated electrograms was not performed.

After circumferential line placement, voltage and pace mapping along the ablation line were used to identify and close gaps. The isolation of all pulmonary veins with bidirectional block was verified with a multipolar circular mapping catheter and was defined as the procedural endpoint.

### Follow-up

After ablation, class I and III antiarrhythmic drugs were not reinitiated. Oral anticoagulation was prescribed for 6 months, and proton pump inhibitors were added for 4 weeks. All patients were followed in the outpatient clinic for 6 months after the ablation. During this follow-up period, 7-day Holter recordings were performed, immediately after the ablation, 3 months and 6 months after the ablation. Additional ECGs and Holter recordings were obtained when patients’ symptoms were suggestive of AF. AF recurrence was defined as a documented AF episode lasting longer than 30 seconds. Early AF recurrence was defined as an AF episode during the first week after the ablation, which is in alignment with previous definitions. [24] This definition was also chosen since continuous Holter monitoring was available for all patients for this time period. Late AF recurrence was defined as any AF episode between 3 and 6 months after the ablation (thus, including a 3-month “blanking period”). All patients with sustained early recurring AF underwent direct current cardioversion. Additional drug administration was left to the discretion of the treating physician.

### Blood Samples

Blood samples were obtained before and 6 months after catheter ablation. Platelet-poor plasma fractions were obtained by centrifugation at 20°C for 10 min at 3500×g, and plasma were stored at −80°C for subsequent analysis.

Plasma levels of hs-CRP were quantified using commercially available specific enzyme-linked immunoabsorbent assay (ELISA, Cusabio Biotech Co., LTD., Newark, USA) according to the manufacturer’s protocol. Results were compared with standard curves and the lower detection limit was 0.08 µg/ml.

### Statistical Analysis

Continuous variables are reported as mean ± one standard deviation or median with interquartile ranges, and categoric variables are reported as frequencies. Continuous variables were compared using Mann-Whitney-U-test (unpaired data), Wilcoxon test (paired data) or according t-tests and categoric variables were compared using the chi-square test.

Multivariable analysis that included variables with a p-value <.1 found in univariate analysis was performed to identify independent predictors of early and late AF recurrence. A p value of <0.05 was considered statistically significant.

## Results

### Rhythm Outcome of Catheter Ablation

Patient characteristics are summarized and compared according to AF type in Table 1. Patients with persistent AF had larger left atrial diameter than patients with paroxysmal AF while other variables including baseline and 6 months hs-CRP were similar. Eight patients (12%) were intermittently on class I or III drugs during follow-up and four patients (6%) were on additional low-dose aspirin.

**Table 1:** Baseline clinical, echocardiographic, and laboratory data of the study population.

	Total n = 68	Paroxysmal AF n = 40	Persistent AF n = 28
Age (years)	59±11	59±4	60±11
Males/females (%)	65/35	65/35	64/36
Lone AF (%)	66	73	57
AF history (months)	72±60	71±62	74±59
Body mass index (kg/m^2^)	29±4	28±4	30±4
Statins (%)	28	28	29
ACEI/ARB (%)	65	63	71
LVEF (%)	60±9	61±7	58±11
LAD (mm)*	43±7	41±6	46±6
hs-CRP (µg/ml) at baseline	2.07±1.10	2.06±1.14	2.06±1.04
hs-CRP (µg/ml) at 6 months	2.14±1.19	2.21±1.37	2.07±0.92

p = .002 for paroxysmal vs. persistent AF; all other variables not significant.

ACEI  =  ACE inhibitor, ARB  =  angiotensin receptor blocker, LVEF  =  left ventricular ejection fraction, LAD  =  left atrial diameter.

Total ablation time was 40±18 min and total energy was 77.279±34.675 J. Complete pulmonary vein isolation as procedural endpoint was achieved in all patients. All but one patient completed the 6-month follow-up including the 7-day Holter ECG.

Early AF recurrence was observed in 38%, while late AF recurrence occurred in 18% of the patients. Patients with recurring early or late AF recurrence are compared to those without recurring AF in Tables 2 and 3. Patients with early AF recurrence had larger left atria while in patients with late recurrence ablation time was longer and ablation energy higher as in patients without recurrences.

**Table 2:** Comparison of baseline clinical, echocardiographic, laboratory and procedural variables between patients with and without ERAF.

	No ERAF n = 42	ERAF n = 26
Age (years)	58±12	62±9
Males/females (%)	69/31	58/42
Lone AF (%)	67	65
Paroxysmal AF/persistent AF (%)	62/38	54/46
AF history (months)	74±63	70±58
Body mass index (kg/m^2^)	28±4	30±4
Statins (%)	29	27
ACEI/ARB (%)	64	69
LVEF (%)	59±9	60±9
LAD (mm)*	42±6	45±6
Total ablation duration (min)	38±17	43±22
Total ablation power (J)	74.030±30.220	84.721±42.743
hs-CRP (µg/ml) at baseline	2.03±1.10	2.11±1.10

* p<0.05; all other variables not significant.

Abbreviations as in Table 1.

**Table 3:** Comparison of baseline clinical, echocardiographic, laboratory and procedural variables between patients with and without AF recurrence after 6 months (please note that one patient did not complete the 6 month follow-up).

	No LRAF n = 55	LRAF n = 12
Age (years)	59±11	61±12
Males/females (%)	62/38	75/25
Lone AF (%)	69	58
Paroxysmal/persistent AF (%)	60/40	50/50
AF history (months)	73±62	68±52
Body mass index (kg/m^2^)	28±4	29±5
Statins (%)	27	33
ACEI/ARB (%)	69	50
LVEF (%)	60±9	56±11
LAD (mm)	43±6	45±9
Total ablation duration (min)*	35±12	61±24
Total ablation power (J)*	67.470±23.733	117.497±44.058
hs-CRP (µg/ml) at baseline	2.09±1.18	2.03±0.61
hs-CRP (µg/ml) at 6 months	2.05±1.11	2.61±1.51
ERAF (%)	35	58

* p<0.001; all other variables not significant.

Abbreviations as in Table 1.

### hs-CRP – Response to Catheter Ablation and Relation with Rhythm Outcome

**Figure 1:**
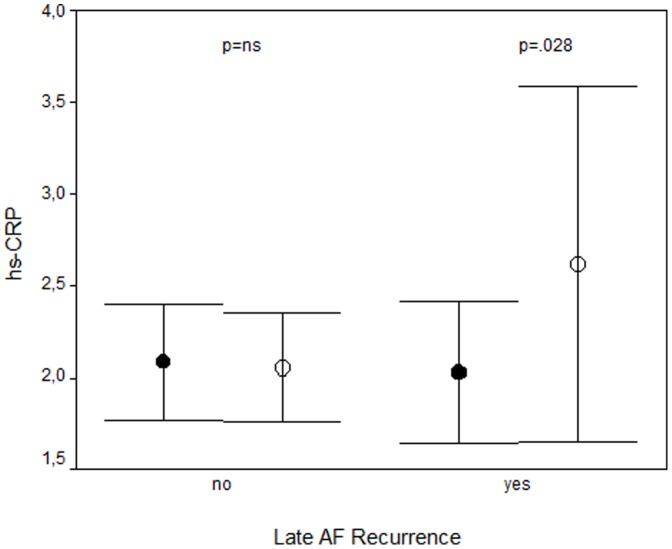
hs-CRP at baseline (•) and after 6 months (○) stratified by rhythm outcome. Please note the similar baseline hs-CRP values between patients with and without recurring AF. In patients without AF recidivism during 6 months follow up hs-CRP remained unchanged, while there was a significant increase in patients with AF recurrences.

Baseline hs-CRP measured 2.07±1.1 µg/ml and was not associated with early or late AF recurrence. At 6 months, hs-CRP levels were comparable with baseline values (2.14±1.19 µg/ml, p = 0.409) and were not different in patients with and without recurring AF (Table 3). However, patients with late AF recurrence showed an hs-CRP increase from 2.03±0.61 to 2.62±1.52 µg/ml (p = 0.028) while hs-CRP remained unchanged in patients without recurring AF (Figure 1). Conversely, patients with an hs-CRP change in the upper tertile (>0.2 µg/ml) had late recurrence in 32% as opposed to 11% in patients in the lower (< −0.3 µg/ml) or intermediate (−0.3–0.2 µg/ml) tertile.

In multivariate analysis including hs-CRP changes, total ablation power and time, total ablation time was the only independent predictor of late AF recurrence (OR 1.088, 95% CI 1.034–1.144, p = 0.001).

## Discussion

### Main Finding

This study demonstrated that baseline hs-CRP was not predictive for early or late AF recurrences after catheter ablation but found an association between hs-CRP increase and late recurring AF. However, ablation time was the only predictor for late AF recidivism.

### Inflammation and AF

Studies of the relationship between AF and inflammation have demonstrated an association between an increased inflammatory activity as measured at the cellular or biochemical level and new-onset or recurring AF. [7] However, it is unclear at present whether initiation of AF induces inflammatory responses or whether the presence of a pre-existing systemic inflammatory state promotes the initiation and persistence of AF. Evidence implicating inammation in the initiation of AF include the increased incidence of AF in the setting of inammatory states such as cardiac surgery or pericarditis, and the observation that baseline CRP levels predict future occurrence of AF. [25] On the other hand, the observation of decreasing CRP levels following restoration of sinus rhythm after persistent [26] or paroxysmal AF [27] supports the notion that inammation is a consequence rather than a cause of AF. It is also likely that both processes feed each other leading to a vicious cycle. In addition the type of AF may also affect this relationship. For instance, previous histologic studies revealed an inflammatory process in the biopsies of patients with long-lasting AF. [28], [29] In contrast, more recent investigations have shown that local inflammation assessed by atrial tissue localization of CRP is more likely involved in paroxysmal rather than in persistent AF. [13]

### Inflammation and Rhythm Outcome of AF Catheter Ablation

There is growing interest on the role of inflammation in recurring AF after catheter ablation. Several findings deserve further discussion: (1) the role of baseline CRP as predictor for AF recurrence is controversial; (2) catheter ablation induces a transient inflammation that may promote early AF recurrences and (3) inflammation may persist but its involvement in late recurrences is unknown.

It has been found that a high baseline hs-CRP level was associated with an abnormal left atrial electrophysiologic substrate and a high incidence of non-pulmonary vein AF sources, and consequently ablation success rate. [12], [20] but this finding has not been consistent. [21] For instance, baseline CRP level predicted early, but not late AF recurrences [19] or was not predictive at all in long-lasting AF. [22] These heterogeneous findings highlight the complexity of this issue that may in part be explained by different study populations, ablation and follow-up strategies.

Isolation of the pulmonary veins with or without creation of additional linear lesions is the cornerstone of today’s AF catheter ablation procedures which has also been applied in our study. Radiofrequency ablation itself creates a localized myocardial necrosis as reflected by an increase in troponin, creatine kinase and activation of the inflammatory cascade. [18] While the inflammatory process is usually considered to occur early after catheter ablation, one previous study has demonstrated a significant rise in CRP at a median of 49 days after AF ablation. [17] This suggests that the inflammatory response may persist for several weeks and could explain recurrences during this follow-up period. In contrast, a decline of hs-CRP level with restoration of sinus rhythm in the non-recurrence group that was accompanied by a reduction in left atrial size has also been reported. These findings suggest that restoration of sinus rhythm by ablation could lead to a decrease of the patients inflammatory state and reverse remodeling. [22]

How does our study add to this body of evidence? While a reduction in CRP with restoration of sinus rhythm suggests inflammation to be the consequence of AF this was not observed in our study. In contrast, we found a CRP increase to be associated with recurring AF but interestingly not between paroxysmal and persistent AF. Since CRP did not change and was not associated with restoration of sinus rhythm with ablation or progression from paroxysmal to persistent AF, we believe that the ablation induced injury with consecutive activation of the inflammatory response may be a contributor to AF recurrences which is also supported by previous studies [17], [18] and our finding of total ablation time being the only predictor of AF recurrence. While this has been speculated for early AF recurrences, the association with late recurrences is – to the best of our knowledge – novel. Nevertheless, we consider this finding hypothesis-generating and believe that further studies incorporating continuous rhythm monitoring and more frequent biomarker measurements are warranted.

### Study Limitations and Implications

This study included a highly-selected patient population, i.e. patients referred for catheter ablation had drug-refractory AF in most cases and was limited to two measurement points, allowing no conclusion regarding the time course of marker changes. In addition, a minority of patients was on antiarrhythmic drugs or aspirin and subsequently a statistical evaluation of drug effects on outcome and CRP was not performed. This analysis did not include other markers of cardiac damage or inflammation. Monitoring of AF recurrence was limited to serial 7-day Holter ECGs and additional ECGs if symptoms suggested AF recurrence which is in line with current guidelines [16] but this strategy may nevertheless have missed asymptomatic AF recurrences. However, CRP levels at 6 months were determined when continuous rhythm monitoring for the preceding 7 days was available.

Despite these limitations, our findings are in agreement with other studies and add to the growing body of evidence linking inflammatory processes and AF pathophysiology. It is tempting to speculate that the frequently observed AF recurrences after ablation therapy may be affected by a transient inflammation, and anti-inflammatory agents could exert favourable effects. Interestingly, various therapeutic agents have the potential to modify CRP levels, including statins, angiotensin converting enzyme inhibitors, angiotensin receptor blockers, steroids as well as antioxidants. [6] Finally, hs-CRP is an easily determined marker in everyday clinical practice and thus, successive measurement of this simple inflammatory index might be clinically useful in monitoring the inflammatory burden in AF. [30]

### Conclusions

Changes in hs-CRP but not baseline hs-CRP are associated with rhythm outcome after AF catheter ablation. This finding points to a link between an inflammatory response and AF recurrence in this setting.
